# Breathing Under Pressure: Psychological Burden and Recovery Trajectories in Patients Receiving Non-Invasive Respiratory Support from Acute COVID-19 to Respiratory Rehabilitation

**DOI:** 10.3390/medsci14020270

**Published:** 2026-05-21

**Authors:** Eleonora Volpato, Valentina Poletti, Maria Luisa de Candia, Lavinia Palma, Alessandro Pilon, Giovanna Elisiana Carpagnano, Paolo Banfi, Paola Pierucci

**Affiliations:** 1 Department of Psychology, Università Cattolica del Sacro Cuore, CAP. 20123 Milan, Italy; valentina.poletti@unicatt.it; 2 IRCCS Fondazione Don Carlo Gnocchi, CAP. 20148 Milan, Italy; 3 Cardiothoracic Department, Respiratory and Critical Care Unit Bari Policlinic University Hospital, Piazza Giulio Cesare 12, CAP. 70100 Bari, Italy; marialuisadecandia@gmail.com (M.L.d.C.); laviniapalma19@hotmail.com (L.P.); alessandro.pilon@gmail.com (A.P.); elisiana.carpagnano@uniba.it (G.E.C.); 4 Section of Respiratory Diseases, Department of Basic Medical Science Neuroscience and Sense Organs, University of Bari ‘Aldo Moro’, CAP. 70124 Bari, Italy; 5 Department of Medicine, Medical School, Libera Università Mediterranea LUM Casamassima Bari 2, Strada Statale 100 Km18, CAP. 70010 Bari, Italy; 6 Respiratory and Bronchoscopy Unit EE Miulli Hospital Acquaviva delle Fonti, Strada Provinciale Acquaviva-Santeramo km 4,100, 70021 Acquaviva delle Fonti, CAP. 70021 Bari, Italy

**Keywords:** non-invasive respiratory support, COVID-19, acute hypoxemic respiratory failure, intensive care unit, physical and rehabilitation medicine, post-traumatic stress symptoms, psychological distress, anxiety

## Abstract

**Background:** Non-invasive respiratory supports (High-Flow Nasal Oxygen, HFNO; Continuous Positive Airway Pressure, CPAP; Non-Invasive Ventilation, NIV) are frequently used in Acute Hypoxemic Respiratory Failure (AHRF). However, the experience of assisted breathing may profoundly affect patients’ psychological balance, particularly during acute critical illness and subsequent rehabilitation. **Aims and objectives**: This longitudinal study investigated the psychological burden associated with non-invasive respiratory support use in patients with COVID-19-related AHRF, exploring changes in psychological functioning from acute hospitalization (RICU/ICU) (T0) to follow-up, conducted at a mean of 6.0 ± 3.1 months after respiratory rehabilitation (T1). **Methods:** Fifty-two patients (mean age = 66.9 ± 9.17 years) were assessed at T0 and T1. Standardized measures evaluated anxiety, psychological distress, post-traumatic stress symptoms, depression, and resilience, in relation to perceived illness severity and subjective experience of non-invasive respiratory support. **Results:** During acute care, patients reported high levels of fear and anxiety related to illness severity and uncertainty. The experience of non-invasive respiratory support, often perceived as a marker of critical condition, was associated with increased fear and anxiety (t(14) = 2.79, *p* = 0.014) compared to the recovery phase, leading to feelings of loss of control and diminished psychological well-being (t(17) = 2.35, *p* = 0.031). However, resilience significantly improved over time (t(16) = −4.78, *p* < 0.001). **Conclusions:** Non-invasive respiratory support may represent a psychologically demanding experience, often perceived as challenging to patients’ sense of safety and control. Encouragingly, psychological adaptation and resilience can improve during rehabilitation. Integrating structured psychological support within respiratory rehabilitation pathways may promote recovery and restore psychological balance in patients requiring assisted ventilation.

## 1. Introduction

In January 2020, a previously unknown contagious respiratory illness was identified in Wuhan, China: severe acute respiratory syndrome coronavirus 2 (SARS-CoV-2), responsible for Coronavirus Disease 2019 (COVID-19) [1]. Since then, COVID-19 has spread worldwide, infecting nearly 800 million individuals and causing approximately 7 million deaths [2]. In Italy alone, around 26 million confirmed cases and almost 200,000 deaths (0.7% case-fatality rate) have been reported, with a higher prevalence among older adults [3,4].

Clinically, patients with mild to moderate COVID-19 commonly present with loss of taste and smell, fever, cough, fatigue, dyspnoea, and sputum production [5]. In some individuals, symptoms may persist over time, leading to the condition known as Long COVID or post-COVID-19 syndrome [6]. In more severe cases, COVID-19 manifests with disabling respiratory symptoms, including marked shortness of breath and breathing difficulties due to excessive mucus production and/or airway collapse [7]. These clinical conditions expose patients to particularly challenging and potentially life-threatening circumstances, increasing mortality risk [8].

Beyond its physical burden, COVID-19 significantly affects psychological well-being [9]. Elevated levels of psychological distress, anxiety and depression have been consistently reported [10], sometimes persisting even after prolonged and demanding rehabilitation processes [11]. Particularly in severe and long-lasting forms of the disease—where structured cardiopulmonary rehabilitation programs become essential [12]—COVID-19 may negatively impact quality of life, restrict autonomy, and alter daily habits [13].

Although most infected individuals experience asymptomatic or mild disease and recover without requiring specific treatment—or receive symptomatic pharmacological management similar to influenza care [14]—a subgroup of patients develops severe acute hypoxemic respiratory failure. In these cases, spontaneous breathing becomes insufficient, requiring respiratory support such as High-Flow Nasal Cannula (HFNC), Continuous Positive Airway Pressure (CPAP), or Non-Invasive Ventilation (NIV), delivered in supine or prone positioning [15].

In patients with COVID-19-associated hypoxemic acute respiratory failure (PaO_2_/FiO_2_ > 150), prolonged awake prone positioning has been adopted to improve oxygenation [16,17]. When respiratory compromise persists, more complex strategies such as NIV are implemented [16,17,18]. NIV consists of delivering mechanical ventilation through a mask interface without the need for endotracheal intubation [19]. Traditionally, NIV has been widely used in acute exacerbations of Chronic Obstructive Pulmonary Disease (COPD) [20] and in chronic respiratory insufficiency related to Obesity Hypoventilation Syndrome (OHS), restrictive thoracic disorders, and neuromuscular diseases [21,22].

However, while non-invasive ventilatory support represents a crucial life-saving intervention, its delivery through a tightly fitted mask covering the nose and mouth—providing intermittent or continuous positive airway pressure—may itself constitute a stressful experience [23]. Although the diverse forms of non-invasive ventilatory support differ in terms of technical characteristics and clinical indication, from the patient’s perspective they may all represent a transition from spontaneous to assisted breathing in a context of perceived threat. In this continuum, non-invasive ventilatory support may be experienced as particularly intrusive due to the use of a fitted mask and the delivery of pressurized airflow. Patients frequently report discomfort related to the mask, describing it as excessively tight and sometimes associated with feelings of claustrophobia or suffocation [24]. Others report difficulty breathing due to the sensation of forced airflow [25,26].

Moreover, hospitalization in Respiratory Intermediate Care Units (RICU) and Intensive Care Units (ICU) is often characterized by fear of the unknown, fear of dying, and feelings of helplessness [27]. The combination of a critical clinical condition, isolation, prone positioning, and continuous respiratory support may therefore amplify anxiety and psychological distress during the acute phase of illness.

Despite the increasing use of non-invasive respiratory supports in the management of COVID-19 [27], research has predominantly focused on the psychological consequences of COVID-19 as a disease, rather than on the psychological experience of respiratory support itself. In particular, the emotional and long-term psychological impact of non-invasive respiratory supports delivered in RICU/ICU settings remains underexplored, especially in relation to patients’ subsequent rehabilitation trajectory and recovery. This gap is clinically relevant, as prolonged hospitalization and respiratory rehabilitation may shape long-term psychological adaptation, resilience, and quality of life.

## 2. Objectives

This study aimed to examine the psychological burden associated with non-invasive ventilatory support in patients with COVID-19-related Acute Hypoxemic Respiratory Failure (AHRF), assessing anxiety, Post-Traumatic Stress Disorder (PTSD) symptoms, psychological distress, and depressive symptoms during RICU/ICU hospitalization and six months after respiratory rehabilitation. Additionally, the study explored how perceived illness severity and the experience of assisted breathing were related to psychological outcomes over time.

## 3. Methods

### 3.1. Ethical Approval

The study was approved by the Ethics Committee of the University Hospital Policlinico of Bari (study number 6847, 24 May 2021). All participants provided written informed consent prior to enrolment. The study was conducted in accordance with the principles of the Declaration of Helsinki.

### 3.2. Design and Treatment Setting

A multicentre, prospective observational pre–post study design was implemented in the following units:

- Respiratory Intensive Care Unit (RICU), Bari Policlinico University Hospital, University of Bari “Aldo Moro”, Bari, Italy;
- Heart–Respiratory Rehabilitation Unit, IRCCS Fondazione Don Carlo Gnocchi, Milan, Italy.

The psychological impact of critical illness and admission to Respiratory Intermediate Care Units (RICU) or Intensive Care Units (ICU) is well documented. Acute stress reactions, distressing psychological experiences, and adverse psychological outcomes following ICU stays have been widely reported. Consequently, psychological assessment and support are increasingly recognized as essential components of comprehensive care.

In line with this evidence, psychological data were collected at two time points: during the acute phase of COVID-19-related AHRF requiring non-invasive ventilatory support (T0, baseline, during RICU stay), and at follow-up after completion of respiratory rehabilitation (T1, after the acute phase, depending on the severity of pulmonary involvement and rehabilitation pathways. The mean follow-up time was 6 ± 3.1 months) to examine psychological trajectories across care settings.

### 3.3. Participants

***Inclusion Criteria***

Participants were eligible if they met all the following criteria:

1. Worsening respiratory symptoms due to severe COVID-19 for ≤ 1 week.
2. Bilateral opacities on chest X-ray consistent with Acute Respiratory Distress Syndrome (ARDS) [28].
3. PaO_2_/FiO_2_ ≤ 300 mmHg.
4. In this study, the term “non-invasive respiratory support “ refers to HFNO, CPAP, and NIV, as commonly used in the management of acute hypoxemic respiratory failure. In particular, we considered: initial treatment for ≥ 12 consecutive hours with High-Flow Nasal Oxygen (HFNO; gas flow ≥ 40 L/min) or Continuous Positive Airway Pressure (CPAP) or Non-Invasive Ventilation (NIV) with Positive End-Expiratory Pressure (PEEP) ≥ 5 cm H_2_O.

Patients undergoing awake prone positioning while receiving non-invasive respiratory support were also included.

***Exclusion Criteria***

Participants were excluded if they:

1. Required Invasive Mechanical Ventilation (IMV) from the onset of respiratory failure;
2. Had incomplete clinical or psychological data for the variables of interest;
3. Had a documented “do not intubate” (DNI) or “do not resuscitate” (DNR) order.

***Study Size***

Statistical power estimation was based on a paired-sample *t*-test (matched pairs), considering a two-tailed significance test, an alpha error probability of 0.05, and a desired power (1 − β error probability) of 0.90. This analysis yielded a non-centrality of the β parameter of 3.31 and a critical t-value of 2.01. Based on these parameters, a minimum total sample size of 44 was determined to achieve the desired statistical power. Anticipating potential attrition or incomplete follow-up data, the target recruitment was increased by approximately 20%.

### 3.4. Measurements

#### 3.4.1. Sociodemographic and Lifestyle Variables

At baseline (T0), the following *sociodemographic and lifestyle variables* were collected: gender, date and place of birth, region of residence, occupation, educational level, marital status, number of children, household composition, smoking status (including average number of cigarettes per day), alcohol consumption (including type and frequency), physical activity (type and frequency), and weight and height for Body Mass Index (BMI) calculation.

#### 3.4.2. Clinical Variables

At baseline (T0), clinical data included: date of COVID-19 symptom onset, date of COVID-19 diagnosis, date of hospitalization, duration of isolation (in days), number of hospitalizations during the previous year (excluding COVID-19-related admission), comorbidities, ongoing pharmacological therapy, and current care setting (ICU or RICU).

At follow-up (T1), recurrence of COVID-19 infection was assessed, including: date of diagnosis, type of infection (asymptomatic, paucisymptomatic/mild, or severe), duration of isolation, treatment setting, and any home-based or outpatient physiotherapy (motor, respiratory, or both). Information regarding ongoing use of non-invasive ventilatory support was also collected (all nights; nights plus several daytime hours; discontinued use).

Additionally, the presence of complications or discomfort related to non-invasive ventilatory support use was recorded, including conjunctivitis; corneal or conjunctival ulcers; gastric distension; aerophagia; skin abrasions or ulceration due to mask contact; nasal or oral dryness; airway obstruction; claustrophobia; pressure sores; or other reported problems. Adoption of awake prone positioning was also documented.

Awake prone positioning refers to the practice of placing spontaneously breathing patients in the prone (face-down) position while receiving oxygen therapy or non-invasive respiratory support (HFNC, CPAP, or NIV), with the aim of improving ventilation–perfusion matching and enhancing oxygenation in the posterior lung regions predominantly affected by COVID-19.

#### 3.4.3. Psychological Measures

Psychological assessment was conducted at baseline (T0, during RICU hospitalization) and at six-month follow-up (T1) using standardized self-report instruments.

-Post-traumatic symptoms were assessed using the *Impact of Event Scale-Revised (IES-R)* [29,30], a 30-item self-report measure evaluating distress related to a specific traumatic event over the previous seven days. The scale includes three subdimensions: Re-experiencing, Avoidance, and Hyperarousal. Items are rated on a 5-point Likert scale (0 = not at all to 4 = extremely). Scores ≥ 24 may indicate clinically significant trauma-related symptoms; while scores ≥ 33 suggest clinically relevant PTSD symptoms. The IES-R is widely used to quantify post-traumatic stress symptoms and to identify individuals who may require specialized psychological support.

-Anxiety symptoms were measured using the *Generalized Anxiety Disorder-7 (GAD-7)* [31], a 7-item self-report questionnaire assessing the severity of generalized anxiety symptoms. Items are rated on a 4-point scale (0–3), yielding total scores ranging from 0 to 21. A cut-off score of 10 has demonstrated good sensitivity (89%) and specificity (82%) for clinically significant anxiety.

-Depressive symptoms were evaluated using the *Patient Health Questionnaire-9 (PHQ-9*) [32,33], a 9-item self-report measure assessing depressive symptom severity. Total scores range from 0 to 27, with scores ≥ 10 indicating clinically relevant depression.

Perceived stress was assessed using the 10-item *Perceived Stress Scale (PSS)* [34], which measures the degree to which individuals appraise situations in their lives as unpredictable, uncontrollable, or overwhelming during the past month. Higher scores indicate greater perceived stress.

-Psychological well-being and health-related quality of life were evaluated using the *Psychological General Well-Being Index (PGWBI)*, a 22-item instrument assessing subjective well-being across multiple domains. Lower scores indicate poorer psychological well-being. A validated short Italian version (PGWB-S, 6 items) [35], referring to the previous month, was also considered.

-State shame and guilt were measured using the *State Shame and Guilt Scale-8 (SSGS-8)* [36], which assesses current feelings of shame and guilt on a 5-point Likert scale.

-Peritraumatic perceptions of fear and life threat were assessed through *visual analogue scales (0–100)* [37], evaluating fear, perceived threat, and perceived likelihood of dying during the most severe phase of COVID-19 illness.

-Resilience was measured using the *Connor–Davidson Resilience Scale (CD-RISC)* [38], a multidimensional instrument assessing adaptive coping and resilience. Items are rated on a 5-point Likert scale. Lower scores reflect reduced resilience and coping resources.

-Illness perception was evaluated using the 9-item *Brief-Illness Perception Questionnaire (B-IPQ)* [39], which assesses cognitive and emotional representations of illness across domains such as consequences, timeline, personal control, treatment control, identity, concern, emotional impact, and illness coherence. The final open-ended item explores perceived causes of the illness.

Finally, an ad hoc questionnaire was developed to explore patients’ subjective experience of non-invasive respiratory support during COVID-19. At the time of the study design, no validated instruments were available to specifically assess this domain. The questionnaire was developed by the research team based on clinical expertise and existing literature on patient experience during respiratory support. It consisted of 11 items, including a combination of closed-ended and open-ended questions, designed to capture both quantitative and qualitative aspects of the experience.

Specifically, the instrument collected information regarding type and duration of respiratory support, prone positioning, communication with healthcare professionals, perceived experience, pain, physical complications, emotional responses (ranked from most to least intense), and the use of a metaphor to describe the experience.

As this instrument was not formally validated, its results were considered exploratory and interpreted with caution. The aim of this tool was to provide a structured yet flexible assessment of patients’ lived experience, rather than a standardized psychometric measure. Completion of the full psychological assessment required approximately 40 min and was supported by a trained psychologist or a respiratory physician during RICU hospitalization and subsequently during rehabilitation.

#### 3.4.4. Statistical Analysis

Statistical analyses were conducted using Jamovi software (version 2.6.45) [40]. All tests were two-tailed, and statistical significance was set at *p* < 0.05.

The primary outcome of the study was post-traumatic stress symptom severity, assessed using the IES-R total score. Secondary outcomes included anxiety, depressive symptoms, perceived stress, psychological well-being, resilience, and illness perception. Descriptive statistics were computed for all study variables. Continuous variables (IES-R, GAD-7, PHQ-9, PSS, PGWBI, SSGS-8, Peritraumatic Perceptions of Fear and Life Threats, CD-RISC, B-IPQ) were summarized using means and standard deviations for normally distributed data, and medians and interquartile ranges where appropriate. Categorical variables (e.g., gender, clinical characteristics, lifestyle variables) were described using absolute frequencies and percentages.

Prior to inferential analyses, assumptions of normality were evaluated using Shapiro–Wilk tests and inspection of Q–Q plots.

To examine longitudinal changes between baseline (T0) and follow-up (T1), paired-sample *t*-tests were performed on global scores of IES-R, GAD-7, PHQ-9, PSS, PGWBI, SSGS-8, CD-RISC, and B-IPQ. Analyses on the primary outcome (IES-R total score) were considered confirmatory within the study design, while analyses on secondary outcomes were considered exploratory. Effect sizes (Cohen’s d for paired samples) were calculated to quantify the magnitude of change over time.

Pearson’s correlation coefficients were computed to assess exploratory associations between post-traumatic stress symptoms (IES-R total and subscales) and other psychological variables. Assumptions of approximate normality were evaluated using Shapiro–Wilk tests and inspection of Q–Q plots. To ensure robustness of the findings, non-parametric correlations (Spearman’s rho and Kendall’s tau) were also computed. As results were consistent across methods, Pearson correlations are reported for clarity. Given the exploratory nature of the study and the relatively small sample size, these analyses should be interpreted with caution. No formal correction for multiple comparisons was applied, and the potential for type I error should be considered when interpreting the results. Due to missing data, the sample size varied across analyses (n range: 42–50 at T0). No imputation procedures were applied. Detailed sample sizes for each analysis are reported in Supplementary Materials S1 and S2.

## 4. Results

### 4.1. Study Sample

Participants were recruited from 95 consecutive patients admitted over a 10-month period in 2022. Of these, 52 patients completed the baseline (T0) psychological assessment. Two patients were subsequently excluded by their treating clinician, resulting in a final baseline sample of 50 participants.

At follow-up (T1), after completion of respiratory rehabilitation, 18 patients (36% of the baseline sample) completed the second assessment. Due to missing data, sample size varied across analyses (T0: n = 40–52; T1: n = 15–48). For each outcome, the sample size corresponds to the number of participants with complete data for the variables included in that specific analysis. No imputation procedures were applied (Supplementary Materials S1 and S2 for details). Participants were classified as responders based on the availability of follow-up data (T1). Baseline comparisons (T0) showed no significant differences between responders and non-responders in post-traumatic stress symptoms, perceived stress, depressive symptoms, anxiety, or resilience. Responders were slightly older and showed higher levels of psychological well-being compared to non-responders (Supplementary Material S3).

Comparative analyses between participants who completed both assessments (T0 and T1) and those lost to follow-up revealed no significant differences in baseline demographic or psychological variables, suggesting the absence of systematic attrition bias.

**Figure 1:**
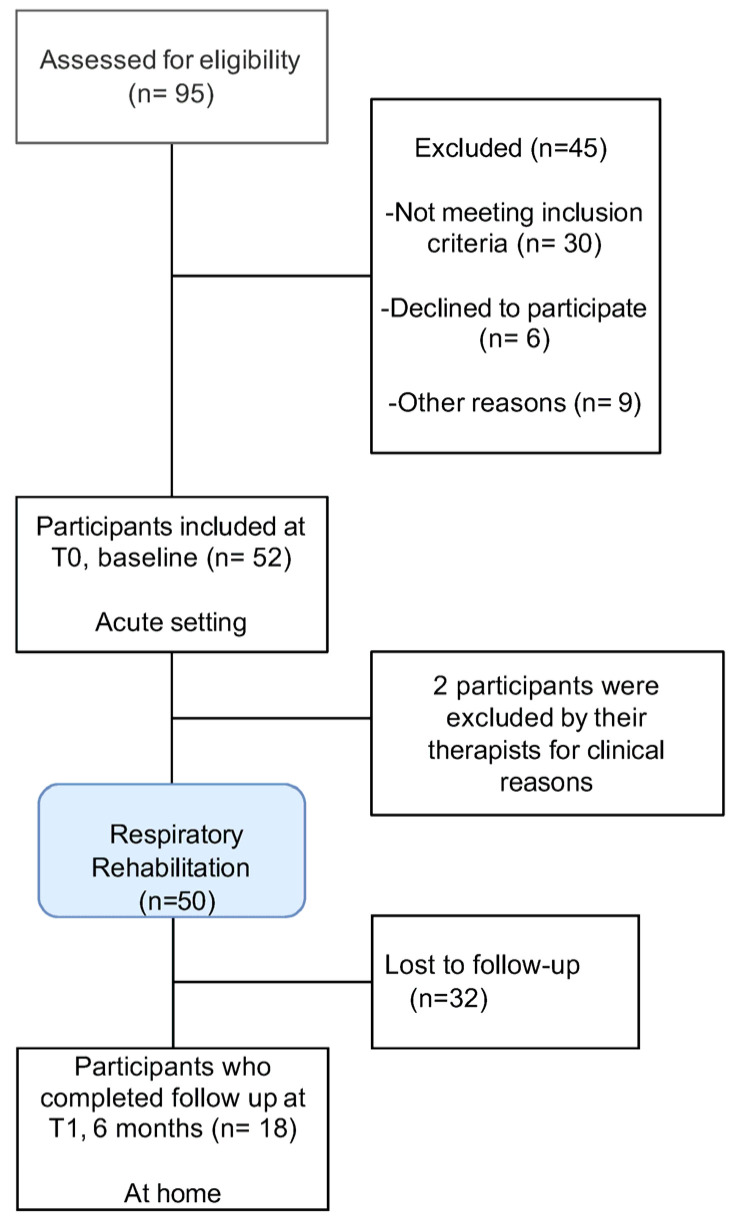
Flow Chart of the participants’ selection.

Details of the enrolment process and participant flow are presented in Figure 1.

### 4.2. Descriptive Data

The baseline sample consisted of 50 participants, of whom 56% were male (n = 28) and 44% female (n = 22). The mean age was 66.9 years (SD = 9.17).

As reported in Table 1, most participants were married (76%, n = 38). Educational levels were distributed as follows: 24% completed Elementary School (n = 12), 30% Lower Middle School (n = 15), and 26% Upper Middle School (n = 13).

Regarding occupational status, most participants were retired (28%, n = 14). Concerning living arrangements, 44% (n = 22) reported living with their spouse.

Regarding lifestyle habits, most participants were either never smokers (52%, n = 26) or former smokers (40%, n = 20).

As reported in Table 2, 48% of participants (n = 24) reported no alcohol consumption, while 38% (n = 19) indicated that they did not engage in any regular physical activity prior to hospital admission.

The mean duration of isolation due to COVID-19 was 27.1 days (SD = 19). The average number of hospital admissions during the previous year, excluding COVID-19-related hospitalization, was 0.76 (SD = 0.64).

At follow-up (T1), 19% of participants (n = 4) reported a recurrent COVID-19 infection. Among these cases, 50% were asymptomatic and 50% presented mild symptoms. The mean duration of isolation was 8.5 days (SD = 2.38).

Regarding post-acute care pathways during the six months following hospitalization, 57% of participants underwent a structured in-hospital motor and respiratory rehabilitation program. The remaining participants did not complete a full inpatient rehabilitation pathway: two patients received outpatient follow-up only, and one patient reported home-based follow-up care.

### 4.3. The Experience with Non-Invasive Ventilatory Support During COVID-19

Participants received different forms of non-invasive respiratory support (HFNO, CPAP, NIV). However, NIV represented the most intensive and psychologically salient form of assisted breathing within the escalation of care. Among participants who completed the follow-up assessment (T1), 55% (n = 10) reported no pain associated with non-invasive respiratory support use during the acute phase. Three participants (16.6%) described non-invasive respiratory support as painful, while the remaining participants did not provide a response to this item.

The most frequently reported physical complications were dryness of the nose and mouth (27.7%, n = 5), followed by conjunctivitis or corneal/conjunctival irritation (22.2%, n = 4), and gastric distension (11.1%, n = 2).

**Figure 2:**
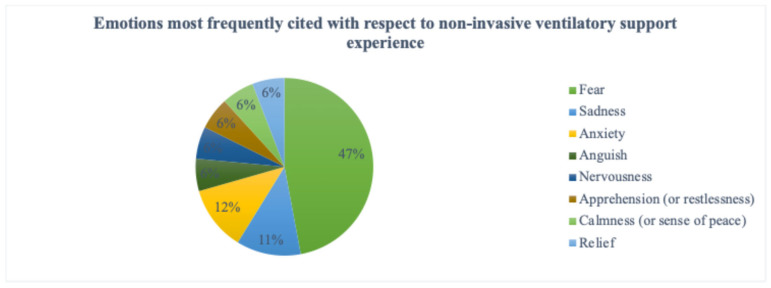
Frequency of the emotions experienced in acute assimilation when using non-invasive respiratory support.

Figure 2 illustrates the frequency of the emotions associated with the experience of non-invasive respiratory support during the acute phase. Fear emerged as the most frequently reported and most intensely perceived emotion. In contrast, disagreement was ranked among the least intense emotions, although both fear (as the primary emotion) and disagreement (as a lower-ranked emotion) were reported by eight participants. A complete hierarchical arrangement of all reported emotions is provided in Supplementary Material S4.

### 4.4. The Relationship Between Variables Before and After Admission for Acute Severe COVID-19

Correlation analyses among baseline psychological variables are reported in Table 3. Non-parametric analyses yielded comparable results (Supplementary Materials S1).

**Figure 3:**
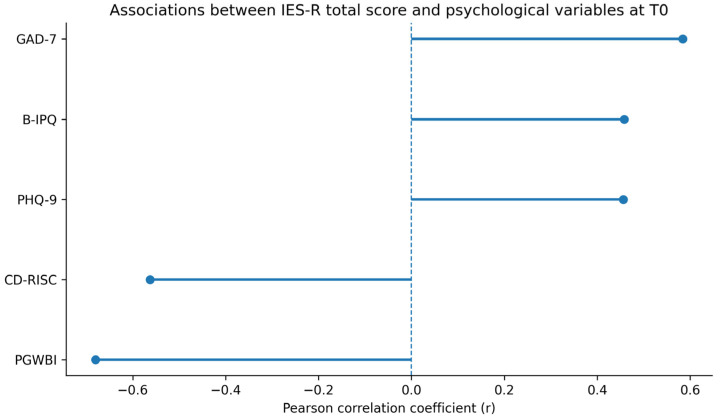
Exploratory baseline associations between post-traumatic stress symptoms (IES-R total score) and selected psychological variables at T0. Error bars represent 95% confidence intervals. Full correlation matrices are reported in Supplementary Materials S1 and S2. Notes: The Impact of Event Scale-Revised (IES-R); Perceived Stress Scale (PSS); The Connor–Davidson Resilience scale (CD-RISC); The Psychological General Well-Being Index-Short (PGWBIs); Patient Health Questionnaire-9 (PHQ-9); Generalized Anxiety Disorder-7 (GAD-7).

At T0, both anxiety and depressive symptoms showed significant associations with multiple indices of post-traumatic stress. Specifically, depressive symptoms were significantly correlated with overall post-traumatic stress (*p* = 0.002), as well as with the IES-R subscales of intrusiveness (*p* = 0.012), avoidance (*p* < 0.001), and hyperarousal (*p* < 0.001). Depression was also significantly associated with illness perception, particularly with the level of illness awareness (*p* = 0.006) (Figure 3).

Similarly, anxiety levels were significantly correlated with total post-traumatic stress (*p* < 0.001) and with all IES-R subdimensions, including intrusiveness (*p* < 0.001), avoidance (*p* < 0.001), and hyperarousal (*p* < 0.001). Anxiety was also strongly associated with illness awareness (*p* < 0.001).

**Figure 4:**
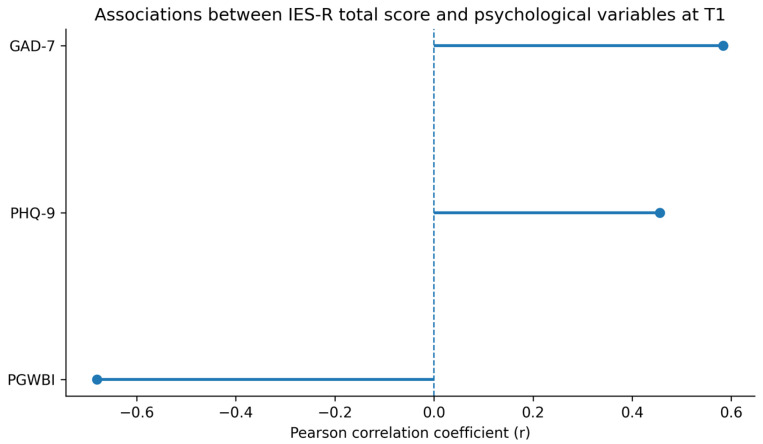
Exploratory follow-up associations between post-traumatic stress symptoms (IES-R total score) and selected psychological variables at T1. Error bars represent 95% confidence intervals. Full correlation matrices are reported in Supplementary Materials S1 and S2. Notes: The Impact of Event Scale-Revised (IES-R); Perceived Stress Scale (PSS); The Psychological General Well-Being Index-Short (PGWBIs); Patient Health Questionnaire-9 (PHQ-9); Generalized Anxiety Disorder-7 (GAD-7).

At follow-up (T1), comparable associations between anxiety, depressive symptoms, post-traumatic stress indices, and illness perception were observed (Figure 4), although the magnitude of these correlations was generally reduced compared to the acute phase.

Paired-sample *t*-test results comparing psychological variables at baseline (T0) and follow-up (T1) are presented in Table 3.

A significant reduction in overall post-traumatic stress symptoms (IES-R total score) was observed at follow-up (t(14) = −2.43, *p* = 0.029, Cohen’s d = 0.63), indicating a moderate effect size. However, no significant changes emerged in the individual IES-R subscales of intrusiveness (t(13) = −0.55, *p* = 0.592), avoidance (t(14) = −0.06, *p* = 0.956), or hyperarousal (t(13) = −0.70, *p* = 0.493).

No statistically significant longitudinal differences were found in anxiety (t(10) = 0.11, *p* = 0.911), depressive symptoms (t(10) = −1.09, *p* = 0.299), perceived stress (t(13) = 1.74, *p* = 0.104), or illness perception (t(14) = 0.31, *p* = 0.759).

In contrast, resilience showed consistent and significant improvement across all CD-RISC subdimensions. Specifically, significant increases were observed in personal competence and tenacity (t(15) = −3.46, *p* = 0.003), trust in one’s instincts and tolerance of negative affect (t(14) = −4.04, *p* = 0.001), positive acceptance of change and secure relationships (t(14) = −4.57, *p* < 0.001), control (t(14) = −2.20, *p* = 0.045), and spiritual influences (t(15) = −4.27, *p* < 0.001). Effect sizes ranged from moderate to large (Cohen’s d = 0.59–1.19), indicating substantial adaptive changes over time.

## 5. Discussion

The present study explored the psychological trajectory of patients who underwent non-invasive respiratory support for COVID-19-related acute hypoxemic respiratory failure, from the acute RICU/ICU phase to the post-rehabilitation period. Our findings suggest that patients receiving non-invasive respiratory support during critical illness reported a clinically relevant psychological distress, particularly in terms of post-traumatic stress symptoms. Although overall PTSD symptoms significantly decreased over time, no parallel reductions were observed in anxiety, depressive symptoms, perceived stress, or illness perception.

Notably, the most consistent longitudinal change concerned resilience, which showed significant and large improvements across all dimensions. This pattern may suggest that psychological recovery, in this context, does not necessarily occur through a simple disappearance of distress symptoms, but rather through a progressive strengthening of adaptive resources. The transition from acute care to rehabilitation may be interpreted as reflecting a process of psychological rebalancing, in which patients gradually regain a sense of control, competence, and acceptance of change, even in the presence of residual emotional vulnerability.

To the best of our knowledge, limited research has investigated the psychological burden associated with the experience of non-invasive respiratory support (HFNC, CPAP, NIV) during acute COVID-19-related respiratory failure and its trajectory following respiratory rehabilitation. The results were consistent with the presence of significant psychological distress during RICU/ICU admission, which partially improved at follow-up.

It is noteworthy that fewer than half of the initial participants completed the second assessment (36% response rate). This attrition may reflect, among other factors, a possible avoidance tendency toward reactivating distressing memories of critical illness. Nevertheless, the response rate observed is consistent with, and in some cases higher than, average participation rates reported in survey-based follow-up studies [41,42].

Although the study involved only two centres, it provides preliminary insight into patients’ lived experiences during a period of sudden and severe respiratory deterioration requiring rapid escalation to non-invasive respiratory support. The sociodemographic profile of the sample—predominantly male, with a mean age of 66.9 years (SD = 9.17), mostly retired and living with a spouse—is consistent with larger epidemiological studies conducted during the pandemic [43,44].

An important clinical aspect concerns the technical conditions under which respiratory support was delivered. In accordance with infection control guidelines during the COVID-19 pandemic, active humidification was generally avoided for CPAP and NIV to reduce aerosol dispersion and protect healthcare workers. Consequently, except for HFNC (which provides built-in humidification), patients received continuous pressurized air without humidification [45]. Under normal circumstances, humidification is recommended to approximate physiological breathing conditions by delivering warmed and humidified air [46,47,48,49]. The prolonged exposure of respiratory mucosa to dry, pressurized air may increase discomfort and contribute to mucosal irritation, thickened secretions, and airway dryness [50].

Consistently, a substantial proportion of participants reported dryness of the nose and mouth (28%) and ocular complications such as conjunctivitis or corneal irritation (22.2%), particularly when full-face interfaces were used. These physical discomforts may have contributed to the subjective experience of respiratory threat, potentially reinforcing the perception of non-invasive respiratory support not only as a life-saving intervention but also as an intrusive and distressing bodily experience.

An additional source of psychological distress may have been the use of awake prone positioning during respiratory support. Awake prone positioning involves placing spontaneously breathing patients in the prone (face-down) position while receiving oxygen therapy or non-invasive respiratory support (HFNC, CPAP, or NIV), with the aim of improving ventilation–perfusion matching and oxygenation in the posterior lung regions most affected by COVID-19 [21,51]. Although clinically beneficial, this position may increase patients’ sense of vulnerability and bodily discomfort, particularly when combined with mask-based ventilatory support.

In line with this interpretation, fear emerged as the most frequently reported emotion associated with the non-invasive respiratory support experience, followed by disagreement and discouragement. These emotional reactions may reflect the sudden deterioration in health status, the abrupt transition to assisted breathing, and an internal alarm response related to perceived threat to life. The experience of struggling to breathe—while physically constrained by a mask interface and, at times, placed in a prone position—may have intensified the perception of loss of control and existential vulnerability.

Interestingly, despite nearly 20% of participants reporting a recurrent COVID-19 infection during the follow-up period, overall PTSD symptoms significantly decreased at T1, while perceived stress remained stable. Most notably, resilience demonstrated significant improvements across all subdimensions, including personal competence and tenacity, trust in one’s instincts and tolerance of negative affect, positive acceptance of change and secure relationships, control, and spiritual influences.

Although only 36% of the original sample completed the follow-up assessment, limiting the generalizability of the findings, the observed increase in resilience is clinically meaningful. These results may suggest not merely symptom reduction, but a possible process of psychological adaptation characterized by enhanced tolerance, acceptance of traumatic experience, and reconstruction of personal coping resources. Importantly, after discharge from acute care, all patients received structured follow-up and, when indicated, were offered respiratory rehabilitation programs. Such integrated post-acute care may be associated with both respiratory recovery and psychological stabilization, although this cannot be established within the present design, facilitating both functional improvement and restoration of internal balance in the aftermath of severe respiratory distress [52,53]. Given the observational design and the absence of a control group, causal relationships cannot be established. The findings should therefore be interpreted as reflecting associations and patients’ subjective experiences over time.

### 5.1. Strengths and Limitations

A key strength of this study lies in its longitudinal design, capturing the psychological trajectory of patients from the acute RICU/ICU phase through post-acute respiratory rehabilitation. To our knowledge, few studies have specifically examined the lived psychological experience associated with non-invasive respiratory support during severe COVID-19-related respiratory failure, integrating both trauma-related symptoms and adaptive psychological dimensions such as resilience. By considering anxiety, depression, perceived stress, PTSD-related symptoms, illness perception, and resilience within the same framework, this study provides a comprehensive view of the psychological burden and adaptation process associated with assisted breathing in critical illness. These findings underscore the importance of integrating structured psychological assessment and support within Respiratory Intermediate and Intensive Care Units, in close collaboration with multidisciplinary healthcare teams. The experience of non-invasive respiratory support appears not only as a physiological intervention but also as a potentially distressing embodied experience, warranting specialized psychological attention.

Several limitations should be acknowledged. First, the sample size was modest, particularly at follow-up (T1), where only 36% of the initial participants completed the second assessment. This attrition limits statistical power and the generalizability of longitudinal findings. Although no significant baseline differences were observed between responders and non-responders, the possibility of self-selection bias cannot be excluded. It is plausible that some patients declined follow-up participation due to avoidance of distressing memories or, conversely, because they no longer perceived a need for medical engagement. An additional limitation is that no subgroup analyses were conducted according to the type of non-invasive respiratory support (HFNO, CPAP, NIV). Given the relatively small sample size and the substantial attrition at follow-up, subgroup stratification would have resulted in limited statistical power and reduced comparability across groups. Future research with larger and more balanced samples is needed to investigate whether different modalities of respiratory support are associated with distinct psychological experiences and trajectories.

Although no significant baseline differences were observed in key psychological distress variables, responders differed from non-responders in age and psychological well-being. This suggests a potential attrition bias, whereby individuals with better perceived well-being may have been more likely to participate in follow-up assessments. Therefore, longitudinal findings should be interpreted with caution.

Second, the study was conducted in two centres, which may limit external validity. Finally, psychological measures relied on self-report instruments, which, although standardized and validated, may be influenced by recall bias and subjective interpretation. Moreover, an additional limitation concerns the use of an ad hoc questionnaire to assess patients’ subjective experience of respiratory support. At the time of the study design, no validated instruments were available to specifically capture the lived experience of non-invasive respiratory support in acute settings. For this reason, a study-specific questionnaire was developed to explore clinically relevant experiential aspects. While this approach allowed the collection of rich and nuanced data, the instrument was not formally validated. Therefore, findings derived from this measure should be considered exploratory and interpreted with caution. This highlights the need for future development and validation of standardized tools in this area.

Despite these limitations, the study offers clinically relevant insights into the psychological impact of assisted ventilation and highlights the need for integrated respiratory and psychological rehabilitation pathways.

### 5.2. Clinical Implications

The findings of this study highlight the need to systematically integrate psychological assessment and support within respiratory and critical care pathways. Non-invasive ventilatory support, while life-saving, may represent an emotionally challenging and embodied experience, characterized by fear, loss of control, and vulnerability. Although different modalities were used, non-invasive ventilatory support may represent the most intrusive and emotionally salient experience, due to mask interface, pressurized airflow, and perceived severity of illness. Healthcare professionals working in RICU/ICU should be aware of the substantial psychological burden associated with assisted breathing during acute respiratory failure. Embedding trained psychologists within respiratory and critical care teams may facilitate early identification of trauma-related symptoms, anxiety, and distress. Brief psychological interventions focused on emotional regulation, sense of control, and meaning making could be incorporated into acute care settings. Furthermore, clear communication regarding the rationale, expected sensations, and benefits of non-invasive respiratory support may help reduce uncertainty and mitigate feelings of helplessness. The significant improvement in resilience observed at follow-up suggests that rehabilitation settings may represent a crucial window for fostering adaptive psychological processes. Respiratory rehabilitation programs could therefore benefit from incorporating structured psychoeducational and resilience-enhancing components alongside physical training. Such integrated models of care may promote not only functional recovery but also restoration of psychological balance following severe respiratory illness. Finally, structured coordination between acute care and rehabilitation services is essential to ensure continuity of care. Establishing clear transitional pathways between inpatient and outpatient settings may facilitate both respiratory and psychological recovery, reducing the risk of long-term distress and improving overall quality of life.

### 5.3. Research Implications

Further longitudinal research is warranted to better understand the trajectory of psychological distress and resilience in patients undergoing non-invasive respiratory support for acute respiratory failure, whether COVID-19-related or due to other etiologies. Future studies should explore how trauma-related symptoms, illness perception, and adaptive resources evolve from the acute phase through rehabilitation and long-term follow-up.

Investigating the interaction between physiological recovery and psychological adaptation may provide valuable insights into how assisted breathing influences both bodily and emotional regulation. Larger multicentre studies are needed to confirm these findings and to identify potential moderators of psychological outcomes, such as prior mental health history, perceived control, or the specific characteristics of respiratory support.

Ultimately, advancing research at the intersection of respiratory medicine and mental health will be essential to develop truly interdisciplinary models of respiratory-based rehabilitation.

## 6. Conclusions

This study highlights the significant psychological burden experienced by patients with severe COVID-19 requiring non-invasive respiratory support for AHRF. Assisted breathing during critical illness appears to represent not only a physiological intervention but also an emotionally demanding experience associated with trauma-related symptoms and distress.

Importantly, although psychological distress did not fully resolve over time, patients demonstrated a meaningful increase in resilience and adaptive capacity following rehabilitation and structured follow-up. These findings suggest that recovery after severe respiratory failure may involve a process of psychological rebalancing rather than simple symptom remission.

Integrating specialized psychological support within respiratory and critical care pathways is therefore essential to address the complex interplay between physiological stabilization and emotional adaptation. Further large-scale longitudinal studies are needed to better understand the long-term evolution of psychological distress and resilience in individuals experiencing severe respiratory failure, regardless of aetiology.

## Figures and Tables

**Table 1:** Socio-demographic information of the sample.

SOCIO-DEMOGRAPHIC DATA	Tot	Male	Female
**Frequency**	50	28 (56%)	22 (44%)
**Age, M (SD)**	66.9 (9.17)	69.3 (2.5)	67.8 (3.4)
**MARITAL STATUS**			
**Married**	38 (76%)	22	16
**Single**	6 (12%)	2	4
**Separated**	1 (2%)	1	0
**Divorced**	2 (4%)	1	1
**Widower**	3 (6%)	2	1
**EDUCATION LEVEL**			
**No school**	3 (6%)	3	0
**Primary school**	12 (24%)	4	8
**Middle school**	15 (30%)	10	5
**College**	13 (26%)	6	7
**Bachelor’s degree**	0	0	0
**Master’s degree**	6 (12%)	4	2
**Post-lauream**	1 (2%)	1	0
**OCCUPATION**			
**Employee**	9 (18%)	8	1
**Freelancer**	3 (6%)	3	1
**Healthcare professional**	2 (4%)	1	1
**Retiree**	14 (28%)	10	4
**Unemployed**	10 (20%)	0	10
**Other**	2 (4%)	0	2
**No answer**	6 (12%)		
**LIVING CONDITION**			
**Living with spouse**	22 (44%)	13	9
**Living with children**	7 (14%)	4	3
**Living with spouse and children**	9 (18%)	4	5
**Living alone**	6 (12%)	4	2
**Living with other relatives**	3 (6%)	1	2
**Living in nursing home**	2 (4%)	1	1
**Living with others (friends)**	1 (2%)	1	0

Notes: M = Mean; SD = Standard Deviation; Tot = Total.

**Table 2:** Principal information of the participants’ lifestyle habits and clinical information.

	Tot	Male	Female
**HEALTH PRACTICES**			
**SMOKING HABIT**			
**Smoker**	4 (8%)	2	2
**Non-smoker**	26 (52%)	10	16
**Ex-smoker**	20 (40%)	16	4
**ALCOHOL CONSUMPTION**			
**No alcohol consumption**	24 (48%)	6	18
**Infrequent alcohol consumption**	12 (12%)	9	3
**Alcohol consumption only at meals**	10 (20%)	9	1
**Alcohol consumption on occasion**	2 (4%)	2	0
**Frequent alcohol consumption**	1 (2%)	1	0
**Very frequent alcohol consumption**	1 (2%)	1	0
**PHYSICAL ACTIVITY**			
**No physical activity**	19 (38%)	6	13
**Infrequent physical activity**	9 (18%)	6	3
**Quite frequent physical activity**	8 (16%)	7	1
**Frequent physical activity**	9 (18%)	7	2
**Very frequent physical activity**	4 (8%)	1	3
**Daily physical activity**	1 (2%)	1	0
**OTHER CLINICAL INFORMATION**			
**Body Mass Index (BMI), M (SD), range per gender**	30.1 (8.2)	~29–31	~28–30
**Height, M (SD)**	169 (8.6)	174 (6.5)	163 (6.6)
**Weight, M (SD)**	81.8 (21.9)	83.3 (18.2)	79.2 (24.1)
**Average days of hospitalization, M (SD)**	27.1 (19)	34.7 (55.5)	18.2 (20)
**Average days between symptoms and diagnosis, M (SD)**	5.20 (5.3)	5.7 (5.5)	4.5 (5)
**Number of hospitalizations during the last year, M (SD)**	0.78 (0.3)	0.76 (0.6)	0.81 (0.7)

Notes: M = Mean; SD = Standard Deviation; Tot = Total.

**Table 3:** Differences between T0 and T1.

									95% Confidence Interval				95% Confidence Interval	
	N (T0)	N (T1)		Statistic	df	p	Mean Difference	SE Difference	Lower	Upper		Effect Size	Lower	Upper
**IES-R_tot**	49	17	Student’s t	−2.4385	14	0.029	−1.9073	0.782	−3.585	−0.2297	Cohen’s d	−0.6296	−1.1767	−0.0641
			Wilcoxon W	22		0.03	−2.21725	0.782	−3.659	−0.145	Rank biserial correlation	−0.6333		
**I_Intrusivity**	47	17	Student’s t	−0.5502	13	0.592	−0.26	0.473	−1.281	0.7609	Cohen’s d	−0.147	−0.6711	0.3825
			Wilcoxon W	48		0.808	−0.265	0.473	−1.5	0.87	Rank biserial correlation	−0.0857		
**A_Avoid**	48	17	Student’s t	−0.0568	13	0.956	−0.0176	0.311	−0.689	0.6533	Cohen’s d	−0.0152	−0.5387	0.509
			Wilcoxon W	46a		1	0.00147	0.311	−0.765	0.815	Rank biserial correlation	0.011		
**H_Hyperarousal**	48	17	Student’s t	−0.7049	13	0.493	−0.5498	0.78	−2.235	1.1352	Cohen’s d	−0.1884	−0.7136	0.3438
			Wilcoxon W	47		0.761	−0.105	0.78	−1.425	1.065	Rank biserial correlation	−0.1048		
**PSS**	43	19	Student’s t	1.7487	13	0.104	5.2857	3.023	−1.244	11.8158	Cohen’s d	0.4674	−0.0938	1.0126
			Wilcoxon W	77.5		0.124	5.00005	3.023	−1.5	12	Rank biserial correlation	0.4762		
**B-IPQ**	49	18	Student’s t	0.3122	14	0.759	1.3333	4.27	−7.825	10.4921	Cohen’s d	0.0806	−0.4277	0.5861
			Wilcoxon W	65.5		0.776	2.23841	4.27	−8	12.5	Rank biserial correlation	0.0917		
**PGWBIs**	49	18	Student’s t	0.7316	14	0.477	1.7333	2.369	−3.348	6.8152	Cohen’s d	0.1889	−0.3252	0.6964
			Wilcoxon W	65 ^a^		0.45	2.00002	2.369	−4	8	Rank biserial correlation	0.2381		
**CD-RISC_tot**	49	18	Student’s t	−6.4	14	<0 .001	−42.2	6.594	−56.342	−28.0577	Cohen’s d	−1.6525	−2.4298	−0.851
			Wilcoxon W	3		0.008	−45.25	6.594	−54.5	−30.5	Rank biserial correlation	−0.95		
**CD1_Personal competence, high standards, and tenacity**	50	18	Student’s t	−3.4648	15	0.003	−10.4375	3.012	−16.858	−4.0167	Cohen’s d	−0.8662	−1.4342	−0.2775
			Wilcoxon W	16.5		0.006	−11.88037	3.012	−17.5	−4	Rank biserial correlation	−0.7574		
**CD2_Trust in one’s instincts, tolerance of negative affect**	52	18	Student’s t	−4.0486	14	0.001	−9.2	2.272	−14.074	−4.3262	Cohen’s d	−1.0453	−1.6685	−0.3986
			Wilcoxon W	8 ^a^		0.001	−10.99999	2.272	−15	−4.5	Rank biserial correlation	−0.8476		
**CD3_Positive acceptance of change, and secure relationship**	49	18	Student’s t	−4.5774	14	<0.001	−8.2667	1.806	−12.14	−4.3932	Cohen’s d	−1.1819	−1.8361	−0.5034
			Wilcoxon W	3.5		0.05	−8.49992	1.806	−12.5	−4	Rank biserial correlation	−0.9417		
**CD4_Control**	49	18	Student’s t	−2.2012	14	0.045	−2.9333	1.333	−5.792	−0.0752	Cohen’s d	−0.5683	−1.1071	−0.0124
			Wilcoxon W	17 ^b^		0.003	−3.99996	1.333	−6.5	0.5	Rank biserial correlation	−0.6264		
**CD5_Spiritual influences**	50	18	Student’s t	−4.279	15	< 0.001	−3.0625	0.716	−4.588	−1.537	Cohen’s d	−1.0697	−1.6783	−0.439
			Wilcoxon W	7.5 ^a^		0.414	−3.49998	0.716	−5	−1.5	Rank biserial correlation	−0.875		
**PHQ-9**	42	17	Student’s t	−1.0964	10	0.299	−2.1818	1.99	−6.616	2.2523	Cohen’s d	−0.3306	−0.9309	0.2851
			Wilcoxon W	19 ^a^		0.893	−1.49997	1.99	−8	3	Rank biserial correlation	−0.3091		
**GAD-7**	42	17	Student’s t	0.114	10	0.911	0.1818	1.594	−3.371	3.7344	Cohen’s d	0.0344	−0.5576	0.6247
			Wilcoxon W	35		< 0.001	0.15417	1.594	−3.371	4	Rank biserial correlation	0.0606		1.594

Notes. H_a_ μ Measure 1 − Measure 2 ≠ 0; ^a^ 1 pair(s) of values were tied; ^b^ 2 pair(s) of values were tied. The Impact of Event Scale-Revised (IES-R); Perceived Stress Scale (PSS); The Connor–Davidson Resilience scale (CD-RISC); The Psychological General Well-Being Index-Short (PGWBIs); Brief-Illness Perception Scale (B-IPQ); Patient Health Questionnaire-9 (PHQ-9); Generalized Anxiety Disorder-7 (GAD-7). Sample size varied across analyses due to missing data (see also Supplementary Materials S1 and S2 for detailed n values).

## Data Availability

The data that support the findings of this study are available on request from the corresponding author.

## Supplementary Materials

The following supporting information can be downloaded at: https://www.mdpi.com/article/10.3390/medsci14020270/s1. Supplementary Material S1: Correlation Matrix at T0. Supplementary Material S2: Correlation Matrix at T1. Supplementary Material S3: Baseline (T0) comparison between responders and non-responders (defined based on availability of follow-up data on the IES-R total score). Supplementary Material S4: Arrangement of the emotions experienced in acute assimilation when using non-invasive ventilatory support, in hierarchical order, from most intense to least intense.
