# Supplementary material for: Breathing Under Pressure: Psychological Burden and Recovery Trajectories in Patients Receiving Non-Invasive Respiratory Support from Acute COVID-19 to Respiratory Rehabilitation
Source: Med Sci (Basel). 2026 May 21;14(2):270. doi: 10.3390/medsci14020270 (PMC13214929; doi:10.3390/medsci14020270)
Supplement: Supplementary file 1 [file medsci-14-00270-s001.zip › Supplementary Material 4.pdf]

Supplementary Material-S4: Arrangement of the emotions experienced in acute assimilation when using the non-invasive ventilatory support, in hierarchical order, from most intense to least intense

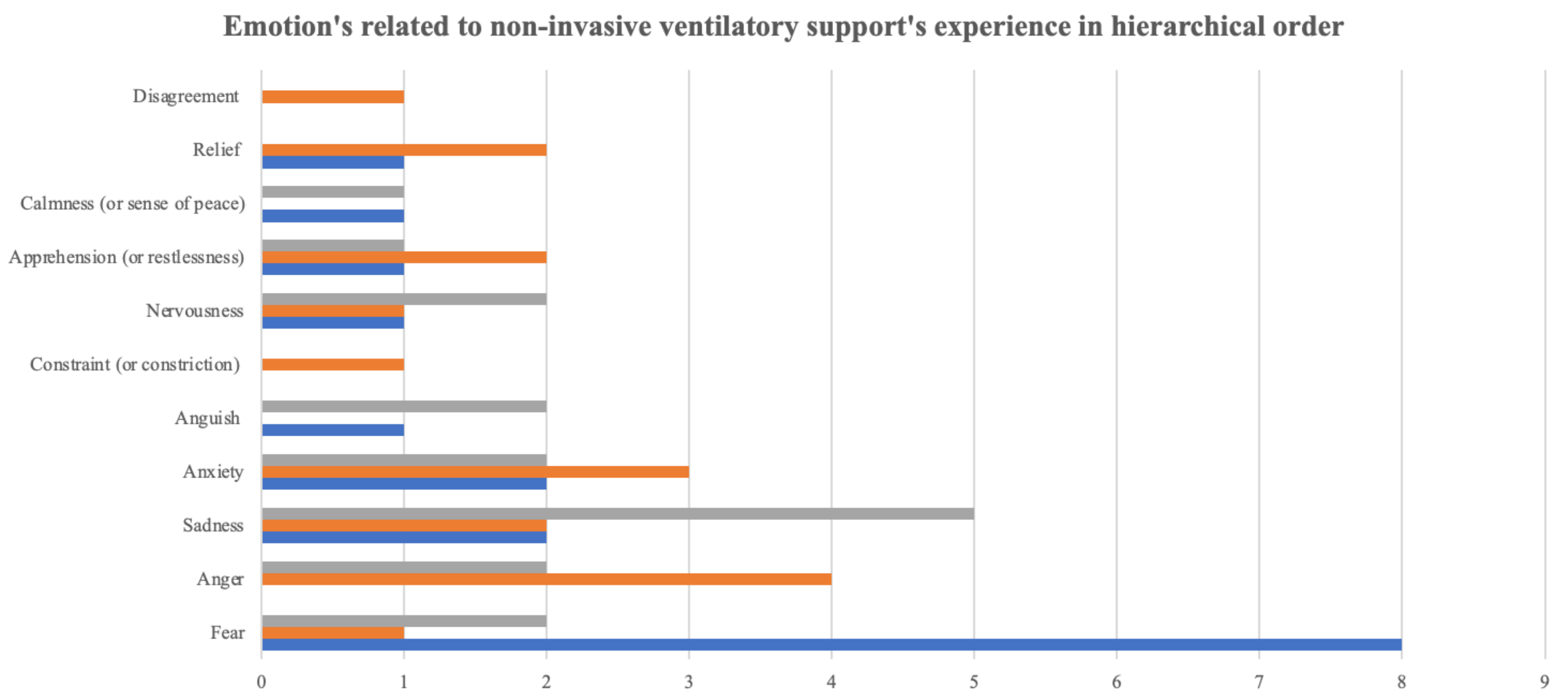

	Fear	Anger	Sadness	Anxiety	Anguish	Constraint (or constriction)	Nervousness	Apprehension (or restlessness)	Calmness (or sense of peace)	Relief	Disagreement
Position_3	2	2	5	2	2	0	2	1	1	0	0
Position_2	1	4	2	3	0	1	1	2	0	2	1
Position_1	8	0	2	2	1	0	1	1	1	1	0

Notes. Each participant was asked to list, in order of importance, from the most (Position 1) to the least (Position 3), the emotions experienced at the impact of the non-invasive ventilatory support for the first time.
